# Early molecular response in East African Philadelphia chromosome-positive chronic myeloid leukaemia patients treated with Imatinib and barriers to access treatment

**DOI:** 10.3332/ecancer.2020.1089

**Published:** 2020-08-18

**Authors:** Oliver Henke, Priscus John Mapendo, Elifuraha Wilson Mkwizu, Philipp le Coutre

**Affiliations:** 1 Institute for Tropical Medicine and International Health, Charité Universitätsmedizin, Berlin, Germany; 2 Cancer Care Centre, Kilimanjaro Christian Medical Centre, Moshi, Tanzania; 3 Medical Department, Division of Hematology, Oncology, and Tumor Immunology (CCM), Charité Universitätsmedizin, Berlin, Germany; a https://orcid.org/0000-0002-9838-9805

**Keywords:** chronic myeloid leukaemia, CML, Tanzania, molecular response, East Africa

## Abstract

**Background:**

Data about haematologic malignancies from Tanzania are sparse. African studies show that chronic myeloid leukaemia (CML) is the most common leukaemia, and registry data display a lower mean age at diagnosis. Prognosis is generally good with tyrosine kinase inhibitors, but the molecular response of Imatinib treatment has never been studied in East Africa, and the outcome remains unknown. This study assessed the early molecular response (MR) as a predictor for long-term outcome and barriers to access treatment.

**Methods:**

A case series of patients with CML from Northern Tanzania documented demographics and laboratory and clinical findings at diagnosis and after 3 months. The regression analysis has been performed on early MR and clinical and demographic variables using the χ2-test. The barriers of potential treatments have been assessed.

**Results:**

A total of 30 patients have been analysed. The mean age was 41 years. All patients had splenomegaly, whereas 16 had hepatomegaly. Complete haematologic response was achieved in 16 and early MR in 9 patients. Hepatomegaly was positively correlated with unfavourable early MR. The average kilometre from home to hospital was 282 km (5–1,158 km). Travel expenses and time investments pose an impediment to treatment.

**Conclusion:**

Patients are younger, and early MR rates are lower compared to other studies. The finding of hepatomegaly as a risk factor for unfavourable early MR was described previously in West Africa. Adherence to therapy is high in the first months of treatment. Furthermore, research is needed to understand the poor MR and the common presentation of hepatomegaly. Outreach clinics might be a solution to reduce impediments to treatment.

## Introduction

According to the Global Cancer Statistics, cancer becomes more of a global public health issue in low- and middle-income countries (LMICs) [1], due to multiple factors, e.g., increased life expectancy leading to the reduction of other causes of death, in addition to advances in diagnostic measurements and detection practices which contribute to higher cancer incidence [2].

For Tanzania, it is estimated that the cancer incidence will double by the year 2030, from 37,000 new cases in 2015 to more than 61,000 cases [3].

### Global incidence of Chronic Myeloid Leukaemia

Chronic myeloid leukaemia (CML) is a haematologic malignancy and belongs to the group of myeloproliferative diseases. CML is the result of the chromosomal translocation t(9;22), which leads to a fusion gene between the *ABL1* proto-oncogene and the *BCR* gene [4]. The resulting product of this fusion gene is an active tyrosine kinase, leading to the uncontrolled proliferation of myeloid cell lineages [5] and, eventually, to leukaemia.

The crude annual incidence of CML in Europe ranges between 0.7 and 1.0/100,000 with a median age at a diagnosis of 57–60 years [6]. The data from the United States of America display an incidence rate of 1.6/100,000, and the WHO suggests that no association with race or ethnicity seems to exist [7]. However, due to the lack of reliable data for LMIC, the data for this part of the world remain an estimation [7], but taking the existing data for extrapolation, the worldwide incidence would be above 100,000 new cases each year.

A study from Sudan states that CML is the most common type of leukaemia in this country in patients with an age group of 16 years and above, whereas ‘leukaemia’ as a group of diseases is the second most common cancer entity in the country [8]. A retrospective data analysis from Ethiopia came to the same conclusion, as CML was the most common leukaemia entity amongst 67 cases of haematologic malignancies at a single centre [9]. CML as a very common entity in haematologic malignancies is also described for Libya in 2012 by Mehdi *et al* [10]. Remarkably, this study reports the fact that Black Africans are significantly more affected than non-Black Libyans.

### Cancer data in Tanzania

At present, only three regional cancer registries in Tanzania exist at Ocean Road Cancer Institute (ORCI) in Dar Es Salaam (Coast Region), Bugando Medical Centre in Mwanza (Lake Region) and Kilimanjaro Christian Medical Centre (KCMC) in Moshi (Kilimanjaro Region). These databases rely mostly on diagnosis made by the respective pathology departments, and hence, haematologic malignancies that are diagnosed by other means such as polymerase chain reaction (PCR), karyotyping, flow cytometry and/or blood smear cytology are not documented. Before this background, the reliability of epidemiologic cancer data and malignant haematologic data, in particular, from Tanzania can be considered as weak as the data presented in the WHO country profile of Tanzania are incidence estimations based on the data of neighbouring countries [11].

### Treatment

The treatment of CML patients changed fundamentally with the development of tyrosine kinase inhibitors (TKI). These drugs lead to greatly extended life expectancy which is now approaching that of the general population. On average, patients lose less than three life years due to CML if regular therapy is available [12]. Four drugs are currently available for the first-line therapy: Imatinib, Nilotinib, Dasatinib and Bosutinib. All of these TKIs are not only very effective but also expensive and a high economic burden for patients [13, 14] in low-resource settings, where healthcare coverage rate is low and out-of-pocket expenditures the norm.

In Tanzania, 3.5 million people are covered by the National Health Insurance Fund (NHIF) and 9.5 million by the Community Health Fund (CHF), which led to a coverage rate of 25% of the population in 2016 [15]. However, neither NHIF nor CHF covers any of the costs for TKI, and the latter does not cover specialised care such as haematologic consultations.

Through MAX foundation, a non-profit organisation in the United States of America, the programme ‘Max Access Solutions’ delivers free Imatinib (Nilotinib, Dasatinib and Ponatinib in the second-/third-line treatment) to partnering institutions in LMIC [16, 17]. This programme was open for patients only at ORCI until 2018 when KCMC Cancer Care Centre (CCC) became the second partnering hospital in Tanzania with a catchment area of 15 million people in the Northern part of the country and the first semi-urban partnering site of the programme worldwide.

Even though CML treatment is available in many LMICs through healthcare coverage or ‘Max Access Solutions’, the knowledge about patients’ characteristics, prognosis, treatment response, adherence to therapy and obstacles to receive medications is limited or unknown for many countries, especially for East African countries.

This study should, therefore, serve two purposes: to display early molecular response to standard treatment with Imatinib as first-line therapy in newly diagnosed East African patients as a predictor for long-term outcome [18–20] and to explore obstacles for receiving treatment amongst these patients.

## Methods

A consecutive case series study was performed on all patients with CML who were enrolled in the ‘Max Access Programme’ between August 2018 and November 2019 at KCMC. Data on demographics and laboratory and clinical findings have been obtained at the time of diagnosis and after 3 months of treatment with Imatinib. The complete haematological response (CHR) was defined according to the European Leukaemia Network (ELN): leucocytes <10^9^/L, basophils <5%, no myelocytes, promyelocytes or blasts in the peripheral blood smear, thrombocytes <450 **×** 10^3^/μL and no palpable spleen [30]. The assessment of early molecular response is based on the international scale (IS) as the ratio of *BCR-ABL1* transcripts to *BCR1* transcripts and is expressed and reported as *BCR-ABL/ABL*% on a log scale, whereby results below 10% are considered as favourable molecular response (fMR) [30]. PCR was performed using ‘GeneXpert® BCR-ABL Ultra’ test from Cepheid®, a quantitative test for *BCR-ABL* major breakpoint (p210) transcripts.

The spleen size was measured from costal margin to the tip of the spleen using a measure tape, and the liver size was obtained by using ultrasound (Kaixin® DCU10) with linear measurements of the right liver lobe (maximum craniocaudal length) in the right medioclavicluar line.

Every patient was seen after 4 weeks for clinic visits and full blood counts, whereby treatment adherence was evaluated by asking the dosing schedule and how many capsules are left. PCR was repeated after 3 months of treatment.

At this time, every patient was asked about obstacles he/she faces with obtaining the treatment. Distance in kilometres from their home to the cancer care centre was determined by using Google Maps. The answers were recorded and categorised accordingly.

Regression analysis was performed on the 3 months molecular response and the clinical/demographic outcome by using *χ*^2^-test and calculation of Fisher´s exact test for *p*-value, acknowledging the small sample size.

All patients gave informed consent to be enrolled into the Max Access Programme. They have been provided with both verbal and written information prior to Imatinib medication and PCR testing. Apart from the questions about barriers to treatment, all data gathered for this study were clinical routine data. Furthermore, written consent was given to use the data for research after explaining the purpose of the data collection, and an assurance was given that enrolment into Max Access Programme is unconditional of the consent of data collection. The continuation of treatment was secured through Max Access Programme.

Ethical clearance has been sought and granted by the Kilimanjaro Christian Medical University College, and ethical principals were applied according to the Declaration of Helsinki.

## Results

In total, 34 patients have been diagnosed with CML between August 2018 and November 2019, of which two patients were lost to follow up after the first and the second months, respectively, and could not be evaluated for three early molecular responses. Another two patients were excluded from this analysis due to a treatment interruption of more than 5 subsequent days because of a temporary stockout of Imatinib.

All 30 analysed patients presented in chronic phase of the disease. Age, sex and laboratory results at diagnosis and 3 months are displayed for each patient in Table 1.

Male:female ratio was 1.14, and the median age at diagnosis was 40.5 (4–60) years. The majority of patients worked as peasants (16), five patients were government employees, three patients were self-employed, two were students and four indicated other occupations. About 26 patients had an annual household income between 1 and 999 US-Dollar.

The median white blood count (WBC) was 300.5 **×** 10^9^/L (78–499 **×** 10^9^/L) and 327.5 **×** 10^3^/μL (115–1004 **×** 10^3^/μL) for thrombocytes, and half of the patients had at least 1% blasts in the peripheral smear at diagnosis. All patients presented with splenomegaly apart from one patient, who underwent splenectomy due to splenomegaly prior to diagnosis. Sixteen patients (53.3%) presented with hepatomegaly.

One patient was HIV positive and used antiretroviral therapy, and none of the patients was on treatment for tuberculosis during the study period.

CHR was achieved by 16 patients, whereas favourable early molecular response by 9 patients.

All patients adhered to the therapy with the dosage prescribed, and every patient was able to repeat the dosage scheme during all visits to the clinic. Missing days of medication occurred in four cases (three patients for 1 day and one patient for 3 days) due to belated attendance to the clinic.

The average kilometres from the patient’s home to CCC were 282 km (5–1158 km). The most common reason mentioned as an impediment for treatment was travel expenses (*n* = 14), followed by time investment for travel (*n* = 13) and missing at work (*n* = 8).

**Figure 1.**
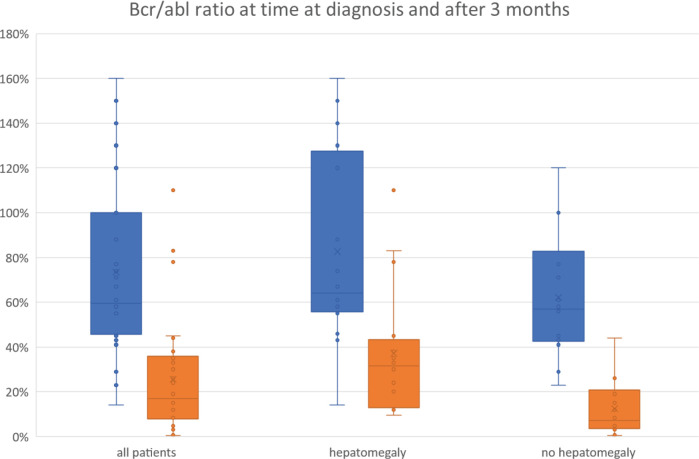
BCR-ABL/ABL ratio at the time of diagnosis and after 3 months of treatment (all patients and by patients with and without hepatomegaly).

Regression analysis (Table 2) showed that CHR significantly correlates with a favourable MR, whereas hepatomegaly is significant negatively correlated with it (Figure 1). Other clinical symptoms as well as sex, occupation and distance from home to hospital were not associated with the molecular outcome. Furthermore, there was no correlation found between the early molecular response results of the patients and the risk scores’ classification of the population (Euro and Sokal, EUTOS and ELTS).

## Discussion

The study was performed to determine the early molecular response amongst Tanzanian CML patients and impediments for treatment. To the best of authors’ knowledge, this is the first study displaying early molecular response amongst CML patients treated with Imatinib in East Africa.

### Demographics

The patients are on average younger than described in Europe and North America. The multicentre ‘IRIS trial’ included 1106 CML patients in chronic phase from Europe, North America and Oceania and reported a median age of 50 years [21]. The cohort had a median age of 39 years (41 years, respectively, if the two paediatric patients are excluded) and accords with data from 2013 from LMIC from 33,985 CML patients [22]. These data derive from the predecessor programme of ‘Max Access Solution’, so called ‘The Glivec® International Patient Assistance Programme’. The findings state a mean average age of African patients of 39.5 years at the time of diagnosis. Only Asian patients were younger with an average mean age of 38.3 years. Across all the studies, men were more often affected than women, and the cohort shows a male/female ratio of 1:14. Mendizabal *et al* [9] stated, in a 2015 analysis of CML data from different regions in the world, that ‘geographic and environmental heterogeneity suggest an important effect of environment’.

Another noticeable finding is the high WBC amongst the patients with an average of 309.9 **×** 10^9^/L. The cohort of the IRIS study had a median WBC in both the study arms of 17.9 and 20.2, respectively. The finding of higher WBC in Sub-Saharan Africa has been described earlier [24–26] and is likely to be a sign of late presentation of the patients to the hospital. Following this logic, the late presentation contributes as well to the fact of splenomegaly in all patients in this study and in the majority of patients in other published African CML data [24,26,27,28].

### Haematologic response

All patients responded to Imatinib therapy, and 53% achieved a CHR after 3 months of therapy according to the criteria of the ELN [29]. Achieving CHR was strongly correlated with an early favourable molecular response as half of these patients had a *BCR-ABL1* ratio ≤10%, whereas none of the patients without CHR achieved a favourable response. The achievement of only 53% is remarkably low if compared to data from ORCI in Dar Es Salaam, where a study reported 91% CHR after 3 months’ treatment with Imatinib in 2016 [30]. However, the patients in the study from Dar Es Salaam had lower WBC at the time of diagnosis. One can assume that patients in Dar Es Salaam, a metropolitan area, present earlier to the hospital due to the better availability of health services. Similar findings were published from Dakar in Senegal with 82.4% CHR [25].

### Early molecular response

The early molecular response rate at 3 months can be described as low with only 30% of patients achieving a *BCR-ABL*/*ABL* ratio ≤10%. On the contrary, the German cohort from the previously cited IRIS study showed that 74.5% (out of 51 patients) achieved a ratio of ≤1% at 3 months of treatment with Imatinib [31], and a recent published study from China stated that 55.7% of 79 patients achieved ≤10% with Imatinib [30]. Adherence to therapy in this study has been secured in all patients, and a significant reduction of WBC, reduction or diminishing of splenomegaly was seen in all the patients. However, an uncertainty remains in the interpretation of the chronic phase (CP) of this cohort. According to the ELN criteria [8], all 30 patients were in CP at the time of diagnosis, but given the fact that no karyotyping was performed—due to the lack of this technique in the routine diagnostic in Tanzania—and additional chromosomal aberrations at diagnosis define the accelerated phase according to the WHO [10]. One could speculate that some patients of this cohort were underdosed with Imatinib and, hence, achieved a poor outcome. Koffi *et al* [28] discovered additional cytogenetic aberrations in 41% of 42 newly diagnosed CML patients from Ivory Coast in 2010. Another interpretation refers to the pharmacokinetics of Imatinib. A study of 126 Nigerian patients [33] revealed that the clearance of Imatinib differs significantly from other publications in different regions of the world, and the authors concluded that this might contribute to the poorer outcome as well. These interpretations, however, remain uncertain amongst East African patients as long as no further studies are conducted to proof these hypotheses.

### Hepatomegaly

From all clinical variables, only hepatomegaly was found to be associated with an unfavourable early molecular response. This finding is in line with other studies from Ivory Coast [26] and Nigeria [34], suggesting that hepatomegaly has a prognostic validity toward worse treatment outcome (significantly reduced overall survival). Furthermore, the study from Dar Es Salaam that described a high CHR of 91% amongst their cohort [30] had only 22.8% of patients with hepatomegaly, whereas, in this cohort, 56.7% had an enlarged liver.

A European study identified hepatomegaly as an adverse predictor of treatment failure: Lekovic et al. analysed 168 CML patients in CP from Serbia and found leucocytosis >100,000 × 10^9^/L, blasts in peripheral blood ≥1%, presence of additional cytogenetic aberrations and hepatomegaly as risk factors for treatment failure [35]. On these findings, the authors developed a prognosis score which correlated better with the treatment outcome of their cohort than the Sokal-, Hasford- and EUTOS-Scores.

Astonishingly, a historic article published in 1978 [36] stated that liver volume has a prognostic value in CML, and furthermore, the hypothesis was discussed that extramedullary haemopoiesis—as the cause of hepatomegaly—in the liver may play an important role in clonal evolution of CML towards a blastic transformation as the authors stated back then. Yet a hypothesis, but a possible explanation for the clinical findings of the previous studies.

### Impediments to treatment

Barriers to accessing treatment in low-resource settings have been researched and published in the last years. A recent qualitative study from Kenya [37] identified the following reasons amongst cancer patients: High costs of testing and treatment, low level of knowledge about cancer in both patients and clinicians, long distances to access diagnostic and treatment and poor communication. CML patients at CCC have the possibility to get a full or partial exemption from diagnostic tests (blood counts, biochemistry and PCR), and the TKI is free of charge through the MAX Access Programme. Apart from these facts, there are still difficulties for the patients. Attendance to the clinics on a regular basis is challenging mostly due to long travel distances and travel fares. The average distance in kilometres from the patient’s home to CCC was 282 km, whereas some patients need to travel more than 1,000 km to have access to TKI. Besides the fare for the travel itself, the necessity to stay overnight before returning to their homes implies further costs. Most of the patients are peasants, and being away from home is tantamount to leave the field and crops unattended. Impassable roads during the rainy season are another challenge for patients to attend on a regular basis. However, the distance has no influence on the early molecular response, which might change during the following years of treatment.

A possible solution is to bring the service to the patients and not vice versa. The ‘Hub and Spoke Model’ [38–40] is an example, whereby a leading centre will supervise smaller health facilities in rural areas for treatment and follow-up of (cancer) patients. Cases needing more attention and sophisticated diagnostic will be referred to the ‘hub’. In CML patients, who are treated with oral medication, this model might be very eligible. It would lead to patients having to visit the leading centre for initial diagnosis and ‘milestones’ during follow-up only, whereas regular clinical and simple laboratory examination and medication refill can be conducted near their homes. In particular, in a territorial state with weak transport infrastructure, it seems to be promising.

### Limitations

The result will not be representative for the population in Tanzania. The location of the hospital will lead to a bias towards urban populations. This bias will be accentuated during the rainy season when patients from rural areas cannot reach the hospital due to impassable roads. Access to KCMC is limited to people with health insurance or financial possibilities to afford expenses for consultation and/or admission. This might lead to the exclusion of many people with a low economic background.

The small sample of patients must be taken into consideration when interpreting the results.

## Conclusions

Imatinib treatment in a semi-urban setting in East Africa is effective; however, the early molecular response amongst the patients is lower than reported in high-income countries. Further studies are needed to explain the low response rate to Imatinib. The role of hepatomegaly as an adverse clinical feature needs to be examined further, especially with regards to extramedullary haematopoiesis and genetic aberrations. The latter might be useful for an adapted African prognosis score and treatment decision-making.

Patients from low-resource countries still face massive barriers despite free treatment and diagnostics. Bringing treatment to the patients—especially in rural areas—might be the key to ensuring good treatment adherence and outcome.

## Conflicts of interest

The authors declare no conflicts of interest.

## Source of funding

PCR testing was supported by funds from the Foundation for Cancer Care in Tanzania.

## Figures and Tables

**Table 1.** Demographic and clinical variables at diagnosis (CHR and *BCR-ABL/ABL* ratio at 3 months).

No.	Age	Sex	WBC (10^9^/L)*	Eosinophils in %	Basophils in %	Blasts in %	Hb (g/dL)*	Thrombocytes (10^3^/μL)*	Hepatomegaly	*BCR-ABL/ABL* ratio*	*BCR-ABL/ABL* ratio at 3 months	CHR at 3 months
1	13	M	384	2	0	0	5.5	479	No	43%	0,63%	Yes
2	42	M	424	4	0	1	7.0	115	No	41%	15%	No
3	53	M	292	5	1	0	9.0	1004	No	23%	4.4%	Yes
4	40	M	366	1	7	0	10.5	320	Yes	58%	45%	No
5	53	M	180	0	1	0	7.2	293	No	100%	5.9%	Yes
6	4	M	499	9	1	0	6.9	198	Yes	67%	35%	No
7	60	F	322	4	0	1	8.7	150	No	56%	26%	Yes
8	36	M	374	3	0	0	5.8	806	Yes	43%	9.6%	Yes
9	31	F	300	0	2	0	13.2	351	No	45%	4.8%	Yes
10	42	M	264	2	1	0	9.8	502	Yes	58%	20%	Yes
11	41	M	225	3	0	2	7.9	309	No	29%	3.8%	Yes
12	50	M	280	5	0	0	8.3	240	Yes	61%	12%	No
13	53	M	390	7	0	0	7.3	398	Yes	14%	13%	Yes
14	30	F	260	5	2	3	9.9	440	Yes	74%	33%	No
15	42	F	78	2	0	4	9.8	278	No	120%	8.4%	Yes
16	54	M	388	1	0	2	8.4	618	Yes#	46%	38%	Yes
17	51	F	240	3	1	1	5.2	280	Yes	58%	83%	No
18	41	F	304	4	0	1	7.1	800	No	58%	12%	No
19	38	F	400	3	0	1	7.1	694	Yes	140%	110%	No
20	33	F	285	2	0	0	7.0	324	Yes	55%	24%	No
21	58	M	448	2	0	0	9.1	800	Yes	120%	78%	No
22	20	M	328	1	2	1	8.6	533	No	61%	44%	No
23	35	F	397	1	0	11	10.0	515	Yes	150%	38%	Yes
24	39	F	274	1	0	0	5.6	213	Yes	130%	30%	No
25	48	F	301	3	0	2	10.1	226	No	56%	3.1%	Yes
26	55	F	227	7	3	7	7.4	618	No	77%	0.57%	Yes
27	34	F	133	4	0	1	5.1	331	Yes	160%	13%	No
28	29	M	267	9	0	1	10.0	172	No	100%	19%	Yes
29	25	F	460	5	0	1	6.9	226	Yes	88%	13%	No
30	29	M	209	4	0	0	10.4	153	No	71%	26%	Yes

All patients had splenomegaly (^#^splenectomy due to splenomegaly before diagnosed with CML); all patients were diagnosed in chronic phase according to ELN criteria; WBC= white blood count; * at time of diagnosis; CHR = complete haematologic response; KM = kilometres; M = male; F = female.

**Table 2.** regression analysis of clinical and sociodemographic variables.

Variable	*n*	fMR at 3 months *n* (%)	ufMR at 3 months *n* (%)	*p*-value (Fisher´s exact test)
All patients	30	9 (30)	21 (70)	
Mean age in years at Diagnosis	39.2			
Gender Male Female	16 14	5 (31.3) 4 (28.6)	11 (68.7) 10 (71.4)	1.00000 OR 1.13636 (0.18298–7.16311)
Employment Agriculture Other	16 14	6 (37.5) 3 (21.4)	10 (62.5) 11 (78.2)	0.43972 OR 2.20000 (0.33914–15.37130)
Kilometres from home to hospital > 30 km > 85 km > 100 km	25 14 12	7 (28) 3 (21) 3 (25)	18 (72) 11 (79) 9 (75)	0.62202 0.43972 0.70356
Complete haematological response yes no	16 14	9 (56.3) 0 (0)	7 (43.7) 14 (100)	**0.00094** OR 0.00272 (0.00004–0.39724)
Clinical symptoms at Dx Splenomegaly Hepatomegaly Skin nodules Fatigue Blasts >1% in peripheral smear Hearing loss/impairment	30^#^ 16 4 13 15 5	- 1 (6,3) 1 (25) 4 (30.7) 4 (26.7) 2 (40)	- 15 (93,8) 3 (75) 9 (69.3) 11 (73.3) 3 (60)	- **0.0043** OR 0.05000 (0.00190–0.57664) 1.00000 1.00000 1.00000 1.00000

fMR = favourable early molecular response (bcr–abl/abl <10%); ufMR = unfavourable early molecular response (*BCR-ABL*/*ABL* > 10%); (^#^1 patient with splenectomy due to splenomegaly before diagnosed with CML)

## References

[1] CDC (2017). Global cancer statistics.

[2] Ferlay J, Soerjomataram I, Dikshit R (2015). Cancer incidence and mortality worldwide: sources, methods and major patterns in GLOBOCAN 2012. Int J Cancer.

[3] International Agency for Research on Cancer and World Health Organisation (2012). GLOBOCAN: estimated cancer incidence, mortality and prevalence worldwide in 2012. https://www.who.int/cancer/country-profiles/tza_en.pdf.

[4] Deininger MW, Goldman JM, Melo JV (2000). The molecular biology of chronic myeloid leukemia. Blood.

[5] Faderl S, Talpaz M, Estrov Z (1993). The biology of chronic myeloid leukemia. N Engl J Med.

[6] Höglund M, Sandin F, Simonsson B (2015). Epidemiology of chronic myeloid leukaemia: an update. Ann Hematol.

[7] WHO (2014). Chronic myelogenous leukamia. Union for International Cancer Control. 2014 review of cancer medicines on the who list of essential medicines. https://www.who.int/selection_medicines/committees/expert/20/applications/CML.pdf.

[8] Weldetsadik AT (2013). Clinical characteristics of patients with hematological malignancies at gondar university hospital, North West Ethiopia. Ethiop Med J.

[9] Saeed MEM, Cao J, Fadul B (2016). A five-year survey of cancer prevalence in Sudan. Anticancer Res.

[10] Mehdi I, Kashmiri AH (2012). Chronic myelogenous leukemia in Libya. South Asian J Cancer.

[11] WHO (2018). International Agency for Research on Cancer. Population Fact Sheets – United Republic of Tanzania. https://gco.iarc.fr/today/data/factsheets/populations/834-tanzania-united-republic-of-fact-sheets.pdf.

[12] Bower H, Bjorkholm M, Dickman PW (2016). Life expectancy of patients with chronic myeloid leukemia approaches the life expectancy of the general population. J Clin Oncol.

[13] Li N, Zheng B, Cai HF (2018). Cost effectiveness and of imatinib, dasatinib, and nilotinib as first-line treatment for chronic-phase chronic myeloid leukemia in China. Clin Drug Investig.

[14] Abboud C, Berman E, Cohen A (2013). The price of drugs for chronic myeloid leukemia (CML) is a reflection of the unsustainable prices of cancer drugs: from the perspective of a large group of CML experts. Blood.

[15] National Health Insurance Fund (2016). http://nhif.or.tz/pages/profile#gsc.tab=0.

[16] Kiarie GW, Othieno-Abinya NA, Riyat MS (2009). The GLIVEC international assistance programme: the Nairobi experience. East Afr Med J.

[17] The Max Foundation (2012). https://www.themaxfoundation.org/our-work/.

[18] Wang R, Cong Y, Li C (2019). Predictive value of early molecular response for deep molecular response in chronic phase of chronic myeloid leukemia. Medicine (Baltimore).

[19] Shanmuganathan N, Hiwase DK, Ross DM (2017). Treatment of chronic myeloid leukemia: assessing risk, monitoring response, and optimizing outcome. Leuk Lymphoma.

[20] Hanfstein B, MC Müller MC, Hehlmann R (2012). Early molecular and cytogenetic response is predictive for long-term progression-free and overall survival in chronic myeloid leukemia (CML). Leukemia.

[21] Hochhaus A, Larson RA, Guilhot F (2017). Long-Term Outcomes of Imatinib Treatment for Chronic Myeloid Leukemia. N Engl J Med.

[22] Mendizabal AM, Garcia-Gonzalez P, Levine PH (2013). Regional variations in age at diagnosis and overall survival among patients with chronic myeloid leukemia from low and middle income countries. Cancer Epidemiol.

[23] Mendizabal AM, Younes N, Levine PH (2016). Geographic and income variations in age at diagnosis and incidence of chronic myeloid leukemia. Int J Hematol.

[24] Tolo DA, Duni S, Clotaire N (2013). Imatinib mesylate effectiveness in chronic myeloid leukemia with additional cytogenetic abnormalities at diagnosis among Black Africans. Adv Hematol.

[25] faye bf, Dieng N, Seck M (2016). Pattern of chronic myeloid leukemia in the imatinib era in a Sub-Saharan African setting. Ann Hematol.

[26] Nanho DC, N’Diaye FS, Tolo A (2008). [Is hepatomegaly a prognosis factor of chronic myeloid leukaemia among African blacks?]. Mali Med.

[27] Entasoltan B, Bekadja MA, Touhami H (2017). Outcome of frontline treatment with “generic” imatinib in adult patients with chronic myeloid leukemia in algerian population: a multicenter study. Mediterr J Hematol Infect Dis.

[28] Koffi KG, Nanho DC, N’dathz E (2010). The effect of imatinib mesylate for newly diagnosed philadelphia chromosome-positive, chronic-phasemyeloid leukemia in Sub-Saharan African patients: the experience of Cˆote d’Ivoire. Adv Hematol.

[29] Baccarani M, Deininger MW, Rosti G (2013). European LeukemiaNet recommendations for the management of chronic myeloid leukemia. Blood.

[30] Tebuka E, Makubi A, Maunda K (2016). Complete haematological response to Imatinib in chronic myeloid leukaemia patients attending the Ocean Road Cancer Institute in Tanzania. Tanzania J Health Res.

[31] Hughes TP, Hochhaus A, Branford S (2010). Long-term prognostic significance of early molecular response to imatinib in newly diagnosed chronic myeloid leukemia: an analysis from the International Randomized Study of Interferon and STI571 (IRIS). Blood.

[32] Cai Z, Jia X, Zi J (2020). BCR-ABL1 transcript decline ratio combined BCR-ABL1ISas a precise predictor for imatinib response and outcome in the patients with chronic myeloid leukemia. J Cancer.

[33] Adeagbo BA, Olugbade TA, Durosinmi MA (2017). Population pharmacokinetics of imatinib in nigerians with chronic myeloid leukemia: clinical implications for dosing and resistance. J Clin Pharm.

[34] Boma PO, Durosinmi MA, Adediran IA (2006). Clinical and prognostic features of Nigerians with chronic myeloid leukemia. Niger Postgrad Med J.

[35] Lekovic D, Gotic M, Milic N (2017). Predictive parameters for imatinib failure in patients with chronic myeloid leukemia. Hematology.

[36] Baccarani M, Zaccaria A, Bagnara GP (1978). The relevance of extramedullary hemopoiesis to the staging of chronic myeloid leukemia. Boll Ist Sieroter Milan.

[37] Makau-Barasa LK, Greene SB, Othieno-Abinya NA (2018). Improving access to cancer testing and treatment in Kenya. J Glob Oncol.

[38] Devarakonda S (2016). Hub and spoke model: making rural healthcare in India affordable, available and accessible. Rural Remote Health.

[39] Elrod JK, Fortenberry JL (2017). The hub-and-spoke organization design revisited: a lifeline for rural hospitals. BMC Health Serv Res.

[40] Asati JS (2018). Implementation of the Hub and Spoke Model to achieve sustainable competitive advantage by health care Global Cancer Care Kenya.

